# The Treating and Filling of Deciduous Teeth*Read before the California State Dental Association, July 1, 1921.—*Pacific Dental Gazette*, Sept., 1922.

**Published:** 1922-12

**Authors:** M. Evangeline Jordon


### THE TREATING AND FILLING OF DECIDUOUS TEETH *

BY M. EVANGELINE JORDON, D.D.S.

Conditions of life are such in this year of 1922 that if a parent wishes his child to have good teeth he must make a constant effort. That such parents are numbered by the thousands is a gratifying sign, but how many communities are prepared with operators who understand the correct handling of children under six years of age, the so-called run-about?

* Read before the California State Dental Association, July 1, 1921.—*Pacific Dental Gazette*, Sept., 1922.

No longer are small children sent home with the advice that "nothing need be done as the teeth are soon to be lost," by the careful dentist who is thinking in terms of 1922 instead of 1900.

Prevention in all lines is the result of conditions revealed by war examinations and a strong light has been thrown upon caries of the teeth as the chief of childhood diseases. As the work in the school clinics has progressed, the need of dental work for the pre-school child has been emphasized. Unfortunately the profession is lagging behind the public in not preparing to handle this great problem—the hundreds of thousands of children between the ages of two and six years needing dental care. The colleges seem satisfied to go on in the same old groove, sending out graduates to repair broken-down teeth, instead of the sensible, painless course of building up perfect dentures that will be a credit to their practice and a priceless possession to the owners for the rest of their lives.

### EFFECT OF FOOD UPON THE TEETH

In the prevention of caries, causes are being studied, and considerable work is being done in animal experiments. In studying my patients from month to month and year to year, from the first, the effect of food has been very apparent. Many babies fed upon artificial foods, overly sweetened and lacking in butter fat present a picture of complete destruction of their teeth by the beginning of the third year, while children breast-fed may show the first sixteen teeth in perfect condition, with complete destruction of the second deciduous molars due to unwise early feeding. This relation is so apparent and the need of so large a lime content for building teeth and bones being necessary, it has been my practice for years to advise limiting the sugars and starches which unfit the appetite for the more necessary milk, dark breads, vegetables and fruits. Many starch eaters refuse milk which they seem unable to digest, but when the sugars are limited they will again take the milk.

In view of the fact that calcification of the fissures of the first permanent molars is usually so imcomplete that with the present-day diet, caries appears before they are fully erupted indicates that more thought must be given to the diet of the pregnant mother.

Two things should be undertaken in the future by thinking dentists. First, the establishing of a standard diet, balanced with relation to the developing teeth; and, second, more education on the evil effects of the other childhood diseases upon the teeth.

### NEED OF EARLIER WORK

The dental hygienists are being trained in a number of colleges to educate the public in mouth hygiene, but there is a wide gap between this work and that of the regular dentist which must be filled by the profession. How many large cities outside of our own state have even one dentist devoting all of his time to the treating and filling of deciduous teeth. Not many, so far as I can learn. You may argue that the work is being done by the local dentist, and perhaps it is, but if what I see of it is a sample of his other work, I wouldn't trust him to work in my mouth. When I open a tooth with a chronic abscess and find the pulp chamber filled with cotton under a silver filling, I thing of whited sepulchres. When even worse cases come in with one filling between two approximal cavities in deciduous molars with exposed pulps under the caries in each cavity, I long to urge every man who does a patch-up, hurry-up job tf that sort to stop and think of the effect of such carelessness not only upon the developing bicuspids beneath, whose enamel organs may be partly destroyed, but of the whole delicately balanced human organization on which, perfectly handcled, means a robust, healthy man, but which careless work in any line may condemn to be a feeble, sickly one. Possibly the perpetrators of such crimes are the men who have urgent business on the outside when papers on children's work are to be read, and who turn the page of the dental magazine when they are published.

I can not impress upon you the urgent need of specially trained children's dentists to fill this gap. The laity are ready; let the profession prepare. Early care of a child enables the operator to watch the development of the face and jaws and correct bad habits which may have such disastrous results in malocclusion. Rogers says, "It is well known that the growth and strength of the alveolar tissue depends upon proper function." Some minor conditions of malocclusion I class under home regulation and teach the patient along that line. Many city children need to be given exercises such as running with the lips tightly closed; and more important still, probably fifty per cent. of children from two to four should be biting hard foods to enlarge the anterior portions of the jaws so that the permanent incisors will have sufficient room. If there is no space between the lower incisors at the third year I advise biting on rubber. "Playing bull dog," the children call it. The father holds a piece of rubber cut from an inner tube, about three inches wide and six inches long and the child tries to pull it away with his teeth. Many times because of small arches one of the upper teeth occludes lingually to the lower teeth and this can be corrected before the tooth is fully erupted by biting upon an orangewood wedge.

### FILLING DECIDUOUS TEETH

At the first appointment I carefully examine the mouth of a child and chart every condition including caries, abscesses, malocclusion and the condition of the gingival margins and the tongue. I do not undertake a case unless I am to put the entire mouth in a healthy condition. It is a great mistake to treat or fill one or two teeth in a child's mouth and let the rest go. It leaves the impression upon the lay mind of the lack of importance of keeping the mouth healthy and the teeth properly functioning. At this time I give an estimate of the approximate time necessary for treatments and filling the teeth, which usually is several weeks and maybe longer, as well as the approximate cost of the work indicated. Before placing any fillings I always use cotton dipped in carbolized resin to pack the cavities and leave it in for two or three days. I usually put cotton into two or three of the largest cavities and at the next appointment the teeth will be separated and the gums will be pushed back, giving a clear view of the cavity. Phenol is a good analgesic and carious deposits may be removed without pain. I may repeat this several times while treating diseased pulps and feel more certain of the health of the tissues left after removing the darkened part under the resin. Copper amalgam is the most practical material for use in the mouths of children. It will not kill the pulp as so many silver amalgam fillings do, and is also valuable for filling small cavities in the permanent molars under certain conditions to be mentioned later. In filling deciduous teeth, only the very small cavities do not need to be extended into compound cavities. The majority of fillings in the deciduous teeth must be cut with a dovetailed step, slightly under-cut. In shaping the cavity cut the step as wide and deep as possible to give strength. The most failures are the breaking of fillings between the step and the approximal portion. Many distal cavities in incisors and cuspids must have a step cut on the lingual surface. The shape of the deciduous molars, the poor enamel strength, and the large pulp with prominent horns, makes a more difficult problem of cavity preparation than most operators realize until they have observed their work for a period of several months. In filling approximal cavities, fill the mesial in the distal tooth, particularly the second deciduous molar first, and then build the distal cavity of the first molar against it, building a wide bridge to prevent food from packing between them. In this way, when the first molar is lost, the second presents a smooth surface to the erupting permanent tooth. If there is the slightest gap, food will crowd in and the acids formed by decomposition will wear away the fillings with great rapidity.

### TREATMENT OF EXPOSED DECIDUOUS PULPS

In the treatment of deciduous pulps every step must be taken with the same degree of care and precision that one would give to a difficult piece of porcelain restoration, if the desired result is to be obtained. This may deter many dentists, because it would seem a waste of time to spend it on temporary teeth which they consider "may soon be lost." This is one of the greatest mistakes of the profession, as the deciduous molars must last from nine to ten years and many show caries in the first few months. When the deciduous molars show a near exposure, within a year of the time it is to be shed and it seems advisable to retain it, I often prepare the cavity as if for a compound filling, cutting a deep step, then cauterize with a saturated solution of nitrate of silver and fill with copper amalgam. This is not to be resorted to except where the tooth is soon to be lost and it must be kept under observation as all near exposures are to be viewed with the suspicion that they are infected and that an abscess may follow. An infected pulp must be treated or the tooth extracted for the sake of the patient's health. It is possible to use pressure anesthesia in some cases but these must be carefully selected. As the rubber dam can not be applied, the saliva in the majority of cases prohibits pressure anesthesia in the mandibular teeth and it is only with easily controlled children that it is practical to use it in the upper teeth. After the removal of the pulp and the wearing off of the effect of the anesthetic pain may appear. Long years of practice have convinced me that devitalizing with some form of arsenous acid is the safest and easiest method. In treating an exposed deciduous pulp, first pack the cavity for twenty-four hours with cotton dipped in carbolized resin.

At the second appointment seal in one devitalizing wafer (The J. Bird Moyer Co.), with pink temporary cement and leave twenty-four hours only. If there is danger of leakage, paint the surrounding tissues with iodin before applying the arsenic. Do not use a rubber dam but keep dry with a folded napkin. Fill the cervical third of the cavity first. If there is an approximal cavity let the cement flow into it for strength.

At the third appointment, with a drill made from a broken cross-cut fissure bur used in the right angle hand-piece, remove the filling. Break down and smooth overhanging walls with a chisel. Remove all caries with a rose bur or spoon excavator and then with a cross-cut fissure bur cut away the roof of the pulp chamber, exposing all root canals. With a rose bur remove all the soft tissue amputating the pulp at the root canals. Under no circumstances use a broach. Seal in a dressing of modified phenol with pink temporary stopping.

At the fourth appointment (two or three days later), remove the modified phenol and dress with formocresol. This coagulates the tissues in the root canals. Seal with pink stopping.

At the fifth appointment, remove the dressing and before the saliva reaches the cavity drop peroxide into the pulp chamber. If there is no effervescence, dry and pack the pulp chamber with alum paste, and seal with white stopping (white indicating that treatment is complete).

Alum paste:

℞. Alum exsic.,
X. Thymol
Glycerol, aa ℥j;
Precip Calc. Phos.
qs. to make stiff paste. M.

At the sixth appointment, remove the outer portion of stopping, being careful not to disturb the part in the pulp chamber. Press the remaining stopping as far as possible into the pulp chamber to force the alum paste into the canals. This compensates for the shrinkage of nerve tissues in the canal. Trim the stopping, leaving as much as possible in the pulp chamber. Then fill the cavity with copper amalgam.

Instruct the parent to prevent the child from putting anything but liquids into the mouth for twelve hours, thus giving the amalgam time to crystallize.

### TREATMENT OF DEAD PULPS

Before treating a deciduous tooth with an abscess, one must determine first, how long the tooth is to be retained, and second, whether the pulp chamber is perforated. It is very important to decide during the second appointment just the treatment to be undertaken as it is neither lucrative, nor satisfactory to the patient, to treat a tooth for weeks and have ultimately to extract it. In treating a deciduous tooth with an abscess, after washing out the tooth, open and leave a day until the soreness has gone. It is necessary only occasionally to lance a badly swollen gum. If this has to be done, paint the area with iodin before lancing and follow the operation by having the child hold water containing an antiseptic in his mouth.

At the second appointment, if the soreness is gone, cut back in the same manner as in removing a pulp. Wash and dry the cavity and seal in formocresol with pink temporary stopping. At the next appointment test with a drop of peroxide. Repeat the treatment with formocresol until all effervescence ceases, then fill with alum paste. If there is a fistula still showing at the third treatment, put into the fistula a wisp of cotton wrapped about a smooth broach, dipped in phenol-sulphonic acid. Carry the point of the broach to the bottom of the fistula, then gently disengage it leaving the cotton to act as a drain. Do not fill the tooth until the gum has healed. It must be remembered that the area around the roots of a treated tooth is a weak spot and after a severe illness an abscess may again form.

After the influenza we had a much larger number of cases to treat over than at any other time since I have limited my work to children. A severe case of whooping cough or measles may have the same result. Two or three years after treatment the patient may return with a fistula on the gum over a treated tooth. In such cases a careful survey will often disclose the nearly erupted bicuspid under the treated tooth, often a year or two before the molar should be shed. It is probable that the bicuspid has had less resistance to overcome in erupting, due to the tissues destroyed at the time of the abscess.

### TREATMENT OF THE FIRST PERMANENT MOLAR

The first permanent molar is the most difficult tooth to save of the permanent teeth because it is so often mistaken for a deciduous tooth and left without care until it is too late to be saved. It is the tooth most frequently affected by hypoplasia and frequently is malformed because of malnutrition before birth or during the child's first year. When there is not actual malformation, the fissures are frequently deep and rough and very susceptible to caries, often softening before the tooth is entirely erupted.

Frequently I dry with alcohol and flow in cement hoping to arrest decay until the occluding molar is in position. There are very few that do not require a filling. Of course the best method of preserving the molar is the gold inlay, but for a long time I hesitated using inlays until root formation was complete. Instead I used the smallest fillings of copper amalgam, but I found that in some cases they would darken the teeth. It is my present practice to use gold inlays as soon as any caries appear. With young children the gold inlays should be inspected monthly because there is a variation in occlusion during the shedding of the deciduous teeth.

I never use silver amalgam. I have seen too many teeth lost by the use of silver amalgam where the least decay left under the filling may cause an exposure of the pulp in the course of two or three years, unsuspected by the parent. Copper amalgam will arrest decay and cause less discoloration. In a few cases I use it as a temporary filling, while educating the child to better hygiene, or tiding him over an illness. If there is a question of approaching a danger spot after all caries is removed, I sterilize with phenol and fill with cement for observation six or eight months before making an inlay. Radiograms are of the greatest assistance in watching the development of these teeth. One of the problems I am still studying is what to do with exposed pulps of these teeth at seven or eight years and perhaps in a few years I may gather enough data to advocate some particular treatment.

### FOLLOW-UP WORK

Very early in my work for children I found that some sort of constant supervision was absolutely necessary, if children were to be saved from suffering, and I began prophylaxis at regular periods. The mere fact of painting the teeth with a disclosing solution, and showing the parent where the teeth have escaped the proper care, has a very salutary effect upon the minds of both the child and the parent. In all my work with children I try to build up an appreciation of the value of perfect teeth, telling them that teeth are more beautiful as well as useful than any jewelry, and praising them when they come for prophylaxis, if they have kept their teeth clean and the gums good and hard but scolding if they have been neglectful. The dental profession has before it the greatest boon that could be conferred upon mankind; the intelligent care and teaching of the laity so that perfect teeth when a child reaches manhood may be the rule and not the exception. This may be accomplished by devising some way of providing every young child with the services of a properly trained children's dentist to supplement the work of the dental hygienist.

### DISCUSSION

*Dr. J. P. Maher*—I am positive that everyone who has listened to this paper by Dr. Jordon appreciates at this time that in handling the children who have come under his observation, he has been very lax in his duties to the child. The parents have every confidence in the dentist and place the child in his care. This trust should not be abused.

Let us now consider the proper attitude of the dentist to his child patients.

From the moment a child is put in his care he becomes morally responsible for the future of that child's mouth. He should realize his responsibility and give that child his very best efforts or refer him to someone who will. The day is long past when the dentist can cooly announce to the mother that these teeth are only baby teeth and will be replaced in a short time by new ones. It is his duty to care for these baby teeth so that the permanent teeth are given every chance to erupt in their normal positions and in a clean, healthy mouth, free from caries.

Wherever possible the work should be of a permanent nature; that is, amalgam fillings or inlays, as we must make every effort to maintain the mesio-distal diameters of the deciduous teeth as the permanent teeth are so much larger than the deciduous teeth. If we do not maintain these dimensions the new teeth can not possibly erupt in their normal positions. I do not mean to find fault with cement fillings as there are many times when it is impossible to do otherwise, and besides cement has a well defined place in children's dentistry.

Oftentimes we find it necessary to extract deciduous teeth before the time that they should be lost. In all such cases we should maintain the space by placing simple retaining appliances on the adjoining teeth.

Another thing we must avoid is having a deciduous tooth of one arch occlude with a permanent tooth of the other arch. If such a condition is allowed to exist the whole articulation will be thrown off and an acquired case of malocclusion will result.

The child's mouth should be examined frequently and between the ages of four and six years we should ascertain if there is room for the permanent teeth to erupt in their normal position. That is, we must recognize whether or not the arch has sufficient lateral width. If the deciduous teeth at this age are perfectly aligned and no developmental spaces exist between the deciduous anterior teeth, it is almost a physical impossibility for the permanent teeth to be in anything but malocclusion.

My advice to every dentist handling children is to consult with an orthodontist frequently—it will do no harm and oftentimes save much trouble in the future.

Dr. Jordon has given us a wonderful paper, not founded on idealistic theories of text-books and magazines, but upon years of experience with children's work. Her methods may not agree with your ideas, but they are the ones that are giving the best results, not over a period of one or two years, but of many years. I have read the paper very carefully and I do not find one thing to criticise. All I can do is to add what I believe the mothers should be taught. The young mother of today is just starving for information that will aid her child in becoming a normal, strong, healthy young man or woman. It is up to the dentist to impart this information.

The mothers should be taught—

1st—The care of the mouth of the infant before the eruption of the baby teeth; that is, to cleanse the mouth before and after every nursing with a small piece of absorbent cotton wrapped around the finger and moistened with a weak solution of boric acid. Also that the baby positively should not be allowed to suck its thumb or should not be allowed the use of pacifiers, as the bony structure of the dental arch during this period is in a state of development and responds very readily to any constant pressure.

2nd—The time of eruption of the baby teeth:

There are twenty in number: eight incisors, four cuspids, and eight molars. The lower centrals begin erupting at about the fifth to the ninth month; the upper central from the eighth to the twelfth month. The laterals and first molars from the twelfth to the eighteenth month. The cuspids from the eighteenth to the twenty-fourth month. The second molars from the twenty-fourth to the thirtieth month. This is the usual rule for healthy, normal babies. If the baby has had any severe illness, or has not been properly nourished, all kinds of variations from the normal are liable to be found.

3rd—During the period of teething everything pertaining to the baby's food should be scrupulously clean, as its vitality, due to pain and fever, is lower during this time and it is much more liable to respond to the injurious effects of unclean nipples, cooking utensils, etc.

4th—The value of the deciduous teeth as related to the child's health and development:

The health and development of the child depends upon its ability to play well, sleep well, and eat well. That the lack of desire to play, eat, and sleep, is induced more by aching and diseased teeth than all other causes combined; the structure and occlusion of the permanent teeth is largely dependent on the preservation, intact, of the deciduous teeth.

5th—The care and preservation of the child's teeth is dependent upon the mother's efforts, for the child will permit the mother to care for them when it would not a stranger. The preservation of a healthy, wholesome mouth condition is the result of a systematic, thorough and regular care carried on in detail.

6th—The surfaces most vulnerable to decay in the child's mouth are the occlusal surfaces of the molars and the approximal surfaces of all the teeth.

7th—As an aid to the proper daily use of the tooth brush, all the surfaces of the teeth should be polished at least once a month with an orangewood stick, pumice or powder, and the approximal surfaces polished with flat floss silk or dental ribbon carrying pumice.

8th—As an aid in the prevention of caries, occlusal surfaces of molars, where the grooves and sulci are deep and imperfectly formed, should be protected by working soft copper cement into them.

9th—Most important of all—the six-year molar is not a temporary tooth—it is not replaced by another tooth if lost, and erupting as it does in early childhood, it must be especially the object of care, and if the grooves and sulci of the occlusal surfaces are deep and perfectly formed they should also be protected by working soft copper cement into them.

10th—The normal development of the jaws preparatory to the eruption of the permanent teeth is dependent largely on the muscles of mastication. For this reason the child should thoroughly masticate all its food, but the food should be prepared in such a way as to require thorough mastication.

11th—According to Dr. Dewey, five out of every nine acquired cases of malocclusion are due to the loss of deciduous tooth structure.

12th—Candy and carbohydrates in other forms are the worst enemies of sound teeth, for the reason that the destruction of the teeth by carries is most easily induced by chemical changes in these substances lodged about the teeth.

13th—The child should not be depended upon to protect its own teeth through its own efforts, but the use of the tooth brush in the hands of the mother is absolutely essential in early childhood.

During the last few years we have listened to many discussions on prevention. But prevention can only be accomplished by the proper care of the deciduous teeth, we look around for authorities on children's dentistry but we search in vain. Prevention is a very popular subject to preach but very unpopular to practice.

Dentistry for children must play a leading part in the health work among the children of our country; remembering that a normal, healthy mouth is the true basis of good health, may we not all be inspired to greater effort in this important cause. A normal mouth with a full complement of clean teeth is a splendid health insurance and an asset to anyone.

Before closing, I would like to thank Dr. Jordon personally for the use of her able paper, and say that just now, after years of work and study, her work is beginning to be appreciated, and as the years go on she will be placed among the real pioneers of better dentistry.

*Dr. Charles A. Sweet*—It is indeed a pleasure to discuss so able a paper as Dr. Jordon has presented, and I hope, when it is published, that everyone will take the opportunity to study it carefully, as I have found it to be a tremendous help in my practice.

I wish that I might criticise this paper, but I must confess that it contains only facts founded on years of experience, and all I can do is offer praise with a few suggestions of other means to accomplish the same result. Considering the filling of deciduous teeth everyone who has used carbolized resin on cotton will appreciate its value. I have used temporary stopping, and on removing it always leaves an irritated gum tissue that makes it almost impossible to dry the cavity, due to the bloody exudate covering the gingival floor. A fact that is sometimes overlooked is the filling of the mesial surface of the distal tooth first, and polishing that filling to the highest degree, so that the erupting tooth has a smooth surface to come in contact with. I am calling this point in Dr. Jordon's paper to your attention again, as I feel that it is very important. The method of devitalizing teeth that Dr. Jordon has given you is the best I have found, and I am truly thankful that I know it, as pressure anesthesia is a complete failure in the child's mouth, unless four walls of tooth structure are standing. In treating putrescent root canals or abscesses on deciduous teeth, I will outline a method that has proved practical in my hands, and which is used in the Forsythe Infirmary and other institutions, with great success: Place cotton rolls in the mouth and dry out the cavity; put a drop of 10 per cent. formalin solution in the cavity, and apply hot air for ten minutes, occasionally adding more of the formalin solution. You may then remove the material in the pulp chamber; then place 4 per cent. formalin solution in cavity and work it about one-third of the way down the root canal, and apply hot air for ten minutes again. This portion of the root canal may be cleaned out and 4 per cent. formalin solution on cotton sealed in with temporary cement; within two days this is removed, and 4 per cent. formalin solution is carried to the end of the root canal, and hot air applied. This is repeated several times, and then it is thoroughly cleaned out. A dressing of eugenol or modified phenol is sealed in for forty-eight hours, and on removing this dressing, if there is no odor, the root canal may be filled. In the Forsythe Infirmary, a microscopic examination is made at this point to determine the presence of bacteria, but in private practice we are unable to do this. To fill the root canals of posterior teeth, use zinc oxide, eugenol and silver nitrate to form a thick paste. In anterior teeth use zinc oxide, eugenol and aristol. This can be forced to place with a pellet of cotton and a cotton root canal point.

In deep decay, where pulps would be exposed by the removal of caries, these pastes may be used, omitting the aristol in the second, with good success.

To treat an exposed pulp of a first permanent molar, place a crystal of thymol at the point of exposure and touch with a hot instrument—this causes the thymol to flow over the exposure and recrystalize—cover this with a thick paste of zinc oxide and eugenol and flow in a hard cement. My experience has not been long enough to determine the ultimate result, but so far it has proven satisfactory.

Before I finish this discussion, I wish to take this opportunity to condemn those dentists who are continually filling deciduous teeth with cement, for I am sure the child would be better off without the teeth filled in that manner, as the cavities would annoy the parent and child until they would be forced to find someone in the community who would place metal fillings that would restore contact and contour and be of real service to the child.

